# Supplementary material for: Physician influence on medication adherence, evidence from a population-based cohort
Source: PLoS One. 2022 Dec 1;17(12):e0278470. doi: 10.1371/journal.pone.0278470 (PMC9714848; doi:10.1371/journal.pone.0278470)
Supplement: S1 File — (DOCX) [file pone.0278470.s001.docx]

## SUPPLEMENTARY APPENDICIES

### Supplementary Appendix A:

### Definitions of variables used to describe physician prescribers.

| **Variable** | **Levels** | **Description** | **Type of measurement** |
| --- | --- | --- | --- |
| Prescriber’s sex | Male/Female | Biologic sex listed in the patient registration file | Time-invariant |
| Prescriber’s country of medical training | Canadian/ Foreign | Country of medical training listed in the physician registration file | Time-invariant |
| Prescriber’s age | Years | Prescriber’s age on the date of each patent’s index date (date of earliest statin dispensation). | Time-varying |
| Prescriber’s years in practice | Years | Number of years between a statin prescriber’s year of medical graduation and a patent’s index date. | Time-varying |
| Prescriber’s remuneration type | Fee-for-service (FFS) / Non fee-for-service (Non-FFS) | Derived from all billing claims submitted by the prescriber (i.e., not only limiting to study patients) between 365 days before and 365 days after the index date of a patient. If at least 80% of these records were coded as FFS claims, the prescriber was deemed a FFS physician for that specific patient. Alternatively, if at least 80% were coded as Non-FFS claims, the prescriber was deemed a Non-FFS physician. Over 95% of GP prescribers could be categorized into one of the groups using this approach. | Time-varying |
| Prescriber’s overall patient count | Number of patients | The number of patients receiving at least one billing claim based on all claims submitted by the prescriber (i.e., not only limiting to study patients) between 365 days before and 365 days after the index date of a patient. | Time-varying |
| Prescriber’s statin patient count | Number of patients | The number of patients with at least one statin dispensation from the GP prescriber. (time-varying) between 365 days before and 365 days after the index date of a patient | Time-varying |

Supplementary Appendix B:

Patient level factors used to control confounding in the assessment of physicians’ influence on statin medication adherence.

| **Variable** | **Levels** | **Description** |
| --- | --- | --- |
| Age | Years | Years since birth to the index date (i.e., the date of the earliest statin dispensation). |
| Sex | Male/female |  |
| Area of residence | Rural/urban | Rural area defined as a population under 1,000 based on linkage between residential postal code and national census data. |
| Household income | Quintiles | Calculated as the mean of the neighborhood mean based on linkage between residential dissemination area code and national census data. |
| Number of distinct prescription medications | Continuous | Defined by anatomical therapeutic chemical classification (ATC) level-5 classes, and measured within 365 days prior to the index date. |
| Number of out-patient visits to GPs | Number | Number of out-patient visits to physicians who were identified as general practitioners (GPs) within 365 days prior to the index date. |
| Number of out-patient visits to specialists | Number | Number of out-patient visits to physicians who were identified as specialists within 365 days prior to the index date. |
| Medication cost paid by government health insurance | Number | Proportion of spending on prescription medications paid by the government health insurance within 365 days prior to the index date. |
| Number of hospitalizations for acute care | Number | Number of hospitalizations for acute care within 365 days prior to the index date. |
| Emergency department visits | Number | Number of visits to emergency department within 365 days prior to the index date. |
| Charlson comorbidity score | 0, 1-2, 3, >3 | Charlson comorbidity index score^1^ measured within 365 days prior to the index date. |
| Continuity of care | Yes/No | Having the same general practitioner (GP) as the most frequently visited GP, the most frequent prescriber of statin, and the complete medical examination provider within 365 days on and after the index date. |
| Calendar year of cohort entry | 2012, 2013, 2014, 2015, 2016, 2017 | Calendar year of the index date (excluded in statistical models as having multicollinearity to prescriber-related variable ‘years in practice’) |
| Chronic conditions | Yes/No | Including osteoporosis, rheumatoid arthritis, hypertension, stroke, ischemic heart disease, acute myocardial infarction, heart failure, multiple sclerosis, Parkinson’s disease, Alzheimer’s disease and dementia, epilepsy, asthma, chronic obstructive pulmonary disease, diabetes, mood and anxiety diseases, schizophrenia, and cancer.  The diagnostic codes and algorithm of these clinical conditions are summarized in a table published by CCDSS.^2^ The conditions were dated back to January 1, 1996. |

###

### Supplementary Appendix C:

### Patient and prescriber characteristics stratified by prescriber’s country of medical training and remuneration type.

| **Characteristics** | Total | Prescriber country of medical training | | Prescriber remuneration type | |
| --- | --- | --- | --- | --- | --- |
|  |  | Canada | Foreign | NFFS | FFS^f^ |
| Patients(n) | n=51,874 | n=15,452 | n=36,422 | n=7,849 | n=44,025 |
| Prescribers (n) | n=1,562 |  |  |  |  |
| Caseload^a^, median (IQR^b^) | 16 (<6, 43) |  |  |  |  |
| % of patients with optimal adherence^c^ | 53.6 | 60.9 | 50.5 | 52.3 | 53.8 |
| **Patient characteristics** |  |  |  |  |  |
| Age, median (IQR) | 59.0 (51.0, 67.0) | 60.0 (52.0, 69.0) | 58.0 (50.0, 67.0) | 59.0 (52.0, 68.0) | 59.0 (51.0, 67.0) |
| Females, n(%) | 22,781 (43.9) | 6,612 (42.8) | 16,169 (44.4) | 3,536 (45.1) | 19,245 (43.7) |
| 1+ hospitalizations for acute care, n(%) | 11,493 (22.2) | 4,911 (31.8) | 6,582 (18.1) | 1,370 (17.5) | 10,123 (23.0) |
| Visits to GPs^d^, median(IQR) | 6.0 (3.0, 9.0) | 6.0 (3.0, 9.0) | 6.0 (3.0, 9.0) | 5.0 (3.0, 9.0) | 6.0 (3.0, 9.0) |
| Visits to specialists, median (IQR) | 2.0 (0.0, 6.0) | 3.0 (1.0, 9.0) | 2.0 (0.0, 5.0) | 1.0 (0.0, 5.0) | 2.0 (1.0, 7.0) |
| 1+ visits to emergency department, n(%) | 10,952 (21.1) | 4,591 (29.7) | 6,361 (17.5) | 1,159 (14.8) | 9,793 (22.2) |
| Income level, n(%) |  |  |  |  |  |
| 1 | 9,569 (18.4) | 2,763 (17.9) | 6,806 (18.7) | 1,766 (22.5) | 7,803 (17.7) |
| 2 | 9,500 (18.3) | 2,678 (17.3) | 6,822 (18.7) | 1,649 (21.0) | 7,851 (17.8) |
| 3 | 9,540 (18.4) | 2,949 (19.1) | 6,591 (18.1) | 1,262 (16.1) | 8,278 (18.8) |
| 4 | 10,685 (20.6) | 3,181 (20.6) | 7,504 (20.6) | 1,672 (21.3) | 9,013 (20.5) |
| 5 | 9,782 (18.9) | 3,098 (20.0) | 6,684 (18.4) | 1,133 (14.4) | 8,649 (19.6) |
| missing | 2,798 (5.4) | 793 (5.1) | 2,005 (5.5) | 367 (4.7) | 2,431 (5.5) |
| Rural residence, n(%)^f^ | 15,830 (30.5) | 4,043 (26.1) | 11,787 (32.4) | 3,735 (47.6) | 12,095 (27.5) |
| Charlson score > 0, n(%) | 16,988 (32.7) | 6,444 (41.7) | 10,544 (29.0) | 2,073 (26.4) | 14,915 (33.9) |
| **Prescribers characterstics^g^** |  |  |  |  |  |
| Age, median (IQR) | 50.0 (40.0, 49.0) | 49.0 (38.0, 59.0) | 50.0 (41.0, 59.0) | 41.0 (35.0, 53.0) | 51.0 (41.0, 60.0) |
| Female, n(%) | 13,532 (26.1) | 5,006 (32.4) | 8,526 (23.4) | 3,012 (38.4) | 10,520 (23.9) |
| Medical training in Canada, n(%) | 15,462 (29.8) |  |  | 1,868 (23.8) | 13,594 (30.9) |
| NFFS^e^ prescriber, n(%) | 7,849 (15.1) | 1,868 (12.1) | 5,981 (16.4) |  |  |
| Years in practice, median (IQR) | 24.0 (13.0, 33.0) | 21.0 (10.0, 33.0) | 24.0 (14.0, 33.0) | 15.0 (9.0, 27.0) | 25.0 (15.0, 34.0) |
| Overall patient count, median (IQR) | 3,346 (2,203, 5,453) | 3,163 (2,022, 4,967) | 3,448 (2,285, 5,619) | 2,112 (1,567, 2,800) | 3,720 (2,409, 5,823) |
| Statin patient count, median (IQR) | 276 (177, 413) | 237 (147, 329) | 303 (188, 440) | 176 (120, 250) | 301 (200, 438) |

^a^Caseload = number of study patients (new statin users) per prescriber; ; ^b^IQR = interquartile range; ^c^Optimal adherence = proportion of days covered >=80% of statin medications; ^d^GP = general practitioners; ^e^NFFS = non-fee-for-service remuneration type; ^f^FFS= fee-for-service remuneration type; ^f^Rural residence = living in areas of a population under 1,000;^g^index date = the date of the first dispensation of a statin medication.

Patient and physician characteristics measured within 365 days prior to the date of the first dispensation of a statin (index date), or on the index date, except that overall patient count, and statin patient count were measured within 365 days prior to and 365 days on and after the index date.

### Supplementary Appendix D:

### Analysis of prescriber years in practice and prescriber age.

The components of prescriber years in practice were associated with optimal medication adherence as described in the main manuscript. However, we excluded prescriber age in this model because of a strong correlation with prescriber years in practice. As a result, we assessed the extent to which prescriber age may have confounded the association between years in practice and adherence to ensure our reported estimates were valid.

To examine the potential confounding by prescriber age, we repeated the analysis of prescriber years in practice within patient strata according to their prescribers’ median age. A prescriber’s median age was calculated from the prescriber’s age on the index date for each patient under their care. The following age categories were constructed: 30-39, 40-49, 50-59, and 60-69.

The within-prescriber effects on years in practice among patient groups stratified by prescriber’s age were consistent with the effect of the whole cohort [prescriber age 30 to 39: aOR = 1.18(0.88, 1.59), 40 to 49: 1.48(1.14, 1.91), 50 to 59: 1.28(1. 24, 1.60), and 60 to 69: 1.46(1.02, 2.09) [See table below]. Similarly, the between-prescriber effects were also consistent with the overall analysis in that the estimated odds of achieving optimal adherence declined with additional years in practice. Of note, the odds ratio estimates were only significant for strata where prescribers’ age was under 50 [prescriber age 30 to 39: aOR = 0.66(0.45, 0.98); 40-49: 0.73(0.55, 0.98).

Odds ratios (95% confidence interval) for the association of every 10 more years in practice with optimal statin adherence stratified by prescriber’s age.

|  |  | Adjusted odds ratio (95%CI)^b^ | |
| --- | --- | --- | --- |
| Median prescriber age^a^ | Number of patients/prescribers | Between prescribers | Within a prescriber |
| 30 to 39 | 11,815/452 | **0.66 (0.45 to 0.98)^c^** | 1.18 (0.88, 1.59) |
| 40 to 49 | 12,964/400 | **0.73 (0.55, 0.98)** | **1.48 (1.14, 1.91)** |
| 50 to 59 | 13,985/299 | 0.89 (0.67, 1.18) | **1.28 (1.24, 1.60)** |
| 60 to 69 | 9,086/215 | 0.90 (0.59, 1.39) | **1.46 (1.02, 2.09)** |

^a^Prescriber’s age = prescriber median age based on all their patient index dates (date the first statin was dispensed); ^b^Adjusted for patient variables including age, sex, urban/rural living, household income level, number of medications by the anatomical therapeutic chemical (ATC) class, number of outpatient visits, percentage of medication cost paid by government health insurance, number of hospitalization for acute care, number of visits to emergency department, Charlson comorbidity score, clinical conditions (osteoporosis, rheumatoid arthritis, hypertension, stroke, ischemic heart disease, acute myocardial infarction, heart failure, multiple sclerosis, Parkinson’s disease, Alzheimer’s disease and dementia, epilepsy, asthma, chronic obstructive pulmonary disease, diabetes, mood and anxiety diseases, schizophrenia, and cancer); also adjusted for prescriber-related variables including prescriber’s sex, country of medical training, remuneration type, overall patient count, and statin patient count; ^c^Odds ratios in bold font are statistically significant (p<0.05); optimal statin adherence = proportion of days covered by statin medications >=80%.

### Supplementary Appendix E:

### De-construction of the prescriber years-in-practice effect (i.e., between-prescriber effect versus within-prescriber effect)

The between-prescriber analysis suggested that the odds of optimal adherence was lower for prescribers with longer years in practice while the within-prescriber estimate indicated the odds of optimal adherence improved during this time. This apparent contradiction was clarified by calculating a dispersion statistic to clarify the within-prescriber result. Dispersion was measured independently for each general practitioner (GP) prescriber as the standard deviation of years in practice (i.e., years in practice was a time-varying measure).^3^ If the standard deviation (i.e., dispersion) was low, it suggested that the prescriber’s new statin patients were clustered within a short period of time during the study period. In contrast, higher standard deviation values (i.e., higher dispersion) suggested that new statin patients were dispersed across the study period. To illustrate the impact of dispersion on the modelling results for optimal adherence, we contrasted optimal adherence with the mean years in practice and the dispersion (or standard deviation of years in practice) to illustrate how these trends co-existed [see table below].

S-Table 3: Percentage of patients exhibiting optimal adherence (proportion of days covered >=80% of statin medications) by quartiles of prescriber’s years in practice, and standard deviation of years in practice.

|  |  |  | Length of years in practice (quartiles) | | | | |
| --- | --- | --- | --- | --- | --- | --- | --- |
|  |  |  | <10.0 years | 10.0 to 19.3 | 19.4 to 31.4 | >31.4 | **Subtotal** |
|  |  | Prescribers (n) | 395 | 386 | 389 | 392 | **1,562** |
|  |  |  | Adherent patients^a^: % (adherent patients / total patients in the group) | | | | |
| **Dispersion^a^ (standard deviation of years in practice when initiating statin for different patients)** | < 0.5 | 390 | 52.8% (281/532) | 44.9%  (257/572) | 49.0%  (249/508) | 46.4%  (187/403) | **48.3% (974/2015)** |
|  | 0.5 to 1.1 | 391 | 53.0% (1528/2883) | 49.7% (1086/2183) | 46.8% (479/1023) | 44.5% (509/1144) | **49.8% (3602/7233)** |
|  | 1.2 to 1.7 | 391 | 55.2% (1552/2811) | 53.5% (2374/4437) | 49.9% (2999/6005) | 52.6% (2834/5389) | **52.3% (9759/18642)** |
|  | > 1.7 | 390 | 57.6% (1505/2611) | 58.0% (2656/4581) | 56.6% (5222/9229) | 53.8% (4069/7563) | **56.1% (13452/23984)** |
|  | **Subtotal** | **1,562** | **55.1% (4866/8837)** | **54.1% (6373/11773)** | **53.4% (8949/16765)** | **52.4% (7599/14499)** | **53.6% (27787/51874)** |

^a^Adherent patient = patients with a proportion of days covered >=80% of statin medications; ^b^standard deviation of years in practice = the standard deviation of a prescriber’s years in paired patients; ^c^adherent patients = patients who presented optimal adherence.

## References of supplementary materials:

1. Quan H, Sundararajan V, Halfon P, et al. Coding algorithms for defining comorbidities in ICD-9-CM and ICD-10 administrative data. *Med Care.* 2005;43(11):1130-1139.

2. CCDSS Case Definitions. <https://health-infobase.canada.ca/ccdss/publication/CCDSS_Case_Definitions_DataCubes_v2018_en.xlsx>. Accessed Jan 1, 2019.

3. Manikandan S. Measures of dispersion. *J Pharmacol Pharmacother.* 2011;2(4):315-316.
