# Supplementary material for: Physician influence on medication adherence, evidence from a population-based cohort
Source: PLoS One. 2022 Dec 1;17(12):e0278470. doi: 10.1371/journal.pone.0278470 (PMC9714848; doi:10.1371/journal.pone.0278470)
Supplement: Attachment — Submitted filename: Review- MYKwak.docx [file pone.0278470.s002.docx]

Reviewer #1: Physician influence on medication adherence, evidence from a population-based cohort.

Authors report an interesting study about physician influence on medication adherence, evidence from a population-based cohort. There are many studies that modeled and verified patient self -management levels and regional characteristics of drug compliance, but there are few studies that analyze the behavioral effects of doctors.

Methods:

It is difficult to conclude because some data are missing such as patient disease duration, prior patient’s disease management education experiences and distribution of comorbidity. It is need to describe some limitation.

In order to prevent unnecessary complications, there were many arbitration and research that combines various behavior theories that allow patients to maintain healthy behavior (like medication compliance). Authors should report some publications.

Also, training counseling experience of physician for compliance can have a positive impact on the patient's behavioral changes. Author should report the indicator (eg. Years in practice in this paper) can use as a proxy indicator for educational skill to improve the self efficacy.

Results:
Some data are missing. (Table1)
- Distribution of different age periods among the older patients: <50 50-64, 65-75, >75 years old.

- Type of comorbidity or diagnostic among patients.

Table2 :

The study outcome was optimal adherence to statin medications during the first

year of therapy, defined by the proportion of days covered (PDC) ≥80%.

Author quantified the influence of GP prescribers for optimal adherence group(independent of patient characteristics) with multivariable logistic regression analyses. But there is no multivariate result for patient group with bad adherence and need to add some table.
