# Supplementary material for: Physician influence on medication adherence, evidence from a population-based cohort
Source: PLoS One. 2022 Dec 1;17(12):e0278470. doi: 10.1371/journal.pone.0278470 (PMC9714848; doi:10.1371/journal.pone.0278470)
Supplement: Attachment — Submitted filename: comments PONE-D-22-22029.docx [file pone.0278470.s003.docx]

Manuscript #: PONE-D-22-22029

**General comments:**

The paper in general is methodologically sound for its internal validity concern and exhibits merit to medication adherence field.

**Specific comments:**

To enhance clarity of the paper, the following issues are advised to be addressed.

Methods

Page 6, paragraph 2 on study design and population:

How did a patient who had multiple statin prescribers over the one-year follow up period was handled? Are there any covariates capturing the cases for multiple prescribers or multiple statin types, especially for the patients who experienced switching of the prescribers, statins, or both were included in the models?

Pages 6-7, outcome measures

Did the dataset contain dosage strength and regimens of the prescribed statins? If that’s so, why not the defined daily doses were used instead of number of tablets for estimating the PDCs.

Page 7, explanatory variables

Apart from demographic characteristics, socio-economic status, and clinical conditions of the study patients, “health literacy” is likely to play an important role on medication adherence. Are there any explanatory variables capturing the patient’s health literacy that were included in the model? It would be good if the paper would distinguish the explanatory variables that classified patient and prescriber characteristics in a systematic way.

Results

Table 1. It would be good if the adherence outcome would be presented in terms of mean +/- SD or median +/- IQR of PDCs in addition to % patients with optimal (≥80% PDCs) adherence.
