# Supplementary material for: Physician influence on medication adherence, evidence from a population-based cohort
Source: PLoS One. 2022 Dec 1;17(12):e0278470. doi: 10.1371/journal.pone.0278470 (PMC9714848; doi:10.1371/journal.pone.0278470)
Supplement: Attachment — Submitted filename: Response to reviewers.docx [file pone.0278470.s004.docx]

**Manuscript #: PONE-D-22-22029**

**General comments:**

The paper in general is methodologically sound for its internal validity concern and exhibits merit to medication adherence field.

**Specific comments:**

To enhance clarity of the paper, the following issues are advised to be addressed.

Methods

**Page 6, paragraph 2 on study design and population:**

**How did a patient who had multiple statin prescribers over the one-year follow up period was handled?**

- Thank you for the comment. The physician listed on the most statin claims during the 1-year follow-up period was identified as the prescriber. We have published information on this procedure previously in PLOS (Yao et al, 2022) demonstrating that a single prescriber can be identified for over 95% of patients. However, we have added another sentence to clarify our procedure for this step
  - *In rare situations where two prescribers were listed on the same number of statin claims, one was selected at random.*

**Are there any covariates capturing the cases for multiple prescribers or multiple statin types, especially for the patients who experienced switching of the prescribers, statins, or both were included in the models?**

- Multiple Prescribers – We have a covariate identifying individuals who visited a specialist in addition to their GP. This variable was included in the model.
- Switching of prescribers – were unable to accurately identify individuals who switched prescribers during the 1-year follow up period. We have added this limitation to the discussion section
  - *Fourth, although the process for identifying the statin prescriber was successful in over 95% of patients, we were unable to identify those who switched prescribers during the follow-up period.*
- Switching between statin products – we allowed switching between statin products without a penalty to adherence score. Allowing switching between drugs of the same class is a conventional approach in the adherence literature.

**Pages 6-7, outcome measures**

**Did the dataset contain dosage strength and regimens of the prescribed statins? If that’s so, why not the defined daily doses were used instead of number of tablets for estimating the PDCs.**

- Our database includes dosage strength and quantity dispensed but does not capture the regimen prescribed by the physician.
- We used tablets per day is a measure we use in Saskatchewan for statins because: a) Statin medications used in Canada are all recommended as once per day administration; b) There is no financial benefit to prescribing a lower dose because the cost is similar for all tablets (e.g., 40mg tablets and 80mg tablets of atorvastatin are the same price). c) All recommended doses are available in our drug plan as single tablets (e.g., atorvastatin 10mg, 20mg, 40mg, and 80mg tablets are available).
- We find DDD to be a useful measure of “exposure” but not “adherence” in epi studies. The disadvantage of the DDD for estimating PDC is due to its fixed dose. For example, the DDD for atorvastatin is 20mg per day. However, 80mg per day is a very commonly prescribed daily dose which equals 4 DDDs (i.e., 4x 20mg). Thus, DDD is highly impacted by the prescribed dose rather than (or in addition to) the number of doses taken in a given time.

**Page 7, explanatory variables**

**Apart from demographic characteristics, socio-economic status, and clinical conditions of the study patients, “health literacy” is likely to play an important role on medication adherence. Are there any explanatory variables capturing the patient’s health literacy that were included in the model?**

- Unfortunately, health literacy measures are not captured in our government’s administrative health claims databases.
- We have added the following sentence to the limitations paragraph in the discussion section:
  - *Also, the databases used for our study lack information about clinical factors (e.g., cholesterol levels), lifestyle factors (e.g., smoking), and some adherence determinants such as health literacy.*

**It would be good if the paper would distinguish the explanatory variables that classified patient and prescriber characteristics in a systematic way.**

- We explained all variables in the supplemental appendix (Appendix A – “Definitions of variables used to describe physician prescribers” and Appendix B – “Patient level factors used to control confounding in the assessment of physicians’ influence on statin medication adherence”). We would be happy to include these tables with the manuscript text if desired.

**Results**

**Table 1. It would be good if the adherence outcome would be presented in terms of mean +/- SD or median +/- IQR of PDCs in addition to % patients with optimal (≥80% PDCs) adherence.**

- Our table stratifies patients by PDC quartiles – thus the IQR are reported in the first row (e.g., <35.7%; 35.7% to 52.2%; 52.3% to 65.4%, and >65.4%).

**Reviewer #1: Physician influence on medication adherence, evidence from a population-based cohort.**

**Authors report an interesting study about physician influence on medication adherence, evidence from a population-based cohort. There are many studies that modeled and verified patient self -management levels and regional characteristics of drug compliance, but there are few studies that analyze the behavioral effects of doctors.**

**Methods:

It is difficult to conclude because some data are missing such as patient disease duration, prior patient’s disease management education experiences and distribution of comorbidity. It is need to describe some limitation.**

- We have added the following sentence in the limitations section (in the discussion)
  - *Also, the databases used for our study lack information about clinical factors (e.g., cholesterol levels), lifestyle factors (e.g., smoking), and some adherence determinants (e.g., health literacy, patient knowledge). Thus, despite including a high number of potential confounders, we were unable to capture some possible confounders and may have only partially addressed others (e.g., specific comorbidities).*

**In order to prevent unnecessary complications, there were many arbitration and research that combines various behavior theories that allow patients to maintain healthy behavior (like medication compliance). Authors should report some publications.**

- We have included two new sentences in the 2^nd^ paragraph of the discussion along with references to ensure the link b/w beliefs/behavior and adherence is clear to the reader.

**Also, training counseling experience of physician for compliance can have a positive impact on the patient's behavioral changes. Author should report the indicator (eg. Years in practice in this paper) can use as a proxy indicator for educational skill to improve the self efficacy.**

- We have re-worded the last sentence prior to limitations (in the discussion) to reflect this suggestion:
  - *It is plausible that frequent prescribing of statin medications throughout the course of a prescriber’s career would improve their skills and experience in positively influencing a patient’s adherence behaviour.*

**Results:
Some data are missing. (Table 1)
- Distribution of different age periods among the older patients: <50 50-64, 65-75, >75 years old.**

**- Type of comorbidity or diagnostic among patients.**

- We measured comorbidity using general measures found in table 1 including the following. We feel these scores distinguish the strata clearly and consistently.
  - Hospitalizations
  - Visits to emergency departments
  - Charlson score
- However, we felt the specific diagnoses of patients would result in a cluttered table that would not add to the interpretation of our results. We hope you agree.

**Table2 :**

**The study outcome was optimal adherence to statin medications during the first**

**year of therapy, defined by the proportion of days covered (PDC) ≥80%.**

**Author quantified the influence of GP prescribers for optimal adherence group(independent of patient characteristics) with multivariable logistic regression analyses. But there is no multivariate result for patient group with bad adherence and need to add some table.**

- Thank you for this comment. We did not report OR’s for patient-level covariables because our modelling approach focused on the physician-related variables only. For that reason, we forced all patient variables in the model and only removed them if they exhibited collinearity with our independent factors of interest. In other words, we built the model to maximize control of confounding for the primary independent variables (i.e., physician factors) at the expense of collinearity between patient factors. Since the patient factors were forced in the model to control for confounding only, we felt their ORs should not be reported in case they were influenced by other factors.
